# Synthesis of Novel Chitin Derivatives Bearing Amino Groups and Evaluation of Their Antifungal Activity

**DOI:** 10.3390/md16100380

**Published:** 2018-10-11

**Authors:** Jingjing Zhang, Fang Luan, Qing Li, Guodong Gu, Fang Dong, Zhanyong Guo

**Affiliations:** 1 Key Laboratory of Coastal Biology and Bioresource Utilization, Yantai Institute of Coastal Zone Research, Chinese Academy of Sciences, Yantai 264003, China; jingjingzhang@yic.ac.cn (J.Z.); qli@yic.ac.cn (Q.L.); fdong@yic.ac.cn (F.D.); 2 University of Chinese Academy of Sciences, Beijing 100049, China; 3 Navigation College, Shandong Jiaotong University, Weihai 264209, China; fluan@yic.ac.cn; 4 Alliance Pharma, Inc., 17 Lee Boulevard, Malvern, PA 19355, USA; guguodong011@gmail.com

**Keywords:** 6-amino-chitin, 3,6-diamino-chitin, antifungal activity, chitin derivative, structure characteristics

## Abstract

Chemical modification is one of the most effective methods to improve the biological activity of chitin. In the current study, we modified C3-OH and C6-OH of chitin (CT) and successfully synthesized 6-amino-chitin (NCT) and 3,6-diamino-chitin (DNCT) through a series of chemical reactions. The structure of NCT and DNCT were characterized by elemental analyses, FT-IR, ^13^C NMR, XRD, and SEM. The inhibitory effects of CT, NCT, and DNCT against six kinds of phytopathogen (*F. oxysporum* f. sp. *cucumerium*, *B. cinerea*, *C. lagenarium*, *P. asparagi*, *F. oxysporum* f. *niveum*, and *G. zeae*) were evaluated using disk diffusion method in vitro. Meanwhile, carbendazim and amphotericin B were used as positive controls. Results revealed that 6-amino-chitin (NCT) and 3,6-diamino-chitin (DNCT) showed improved antifungal properties compared with pristine chitin. Moreover, DNCT exhibited the better antifungal property than NCT. Especially, while the inhibition zone diameters of NCT are ranged from 11.2 to 16.3 mm, DNCT are about 11.4–20.4 mm. These data demonstrated that the introduction of amino group into chitin derivatives could be key to increasing the antifungal activity of such compounds, and the greater the number of amino groups in the chitin derivatives, the better their antifungal activity was.

## 1. Introduction

Chitin, with the chemical structure of β-(1-4)-linked 2-acetamido-2-deoxy-d-glucopyranose units, is the most abundant nitrogen-bearing compound in nature and the second most abundant biopolymer on earth after cellulose. Chitin can be achieved from the exoskeletons of arthropods such as crabs, shrimps, lobsters, prawns, or the cell walls of some microorganisms and some fungi [1,2,3,4,5]. As a natural renewable resource, chitin possesses advantages in unique physicochemical characteristics and bioactivities expressed as its biodegradability, biocompatibility, and non-toxicity property [6,7,8,9].

Considerable attention has been paid to the antimicrobial activity of chitin and its derivatives in recent years [10,11,12]. Particularly, many studies have explored modification routes aimed at the 6-OH of chitin which are targeted to specific applications [13,14]. For example, the preparation of tosyl-chitin intermediates through the tosylation of the 6-OH group of chitin has been reported since the tosyl group is a good electrophile and leaving group. However, because of the intramolecular hydrogen bonds with neighboring repeating units as well as the steric hindrance resulted from neighboring acetamide groups, the 3-OH group of chitin has the lower reactivity compared with 6-OH. Consequently, studies based on 3-OH substitution route for the preparation of chitin derivatives have been less explored.

It has been reported that the presence of the amino groups in polysaccharide enables distinctive biological functions, such as anticoagulant activity, antibacterial property, and antioxidant activity [15,16,17]. Meanwhile, it was found that the antifungal activity of carbohydrate derivatives against the plant pathogenic fungi increased with an increase in active amino groups substituent [18,19]. Inspired it, amino can be immobilized on chitin at the 3-OH and 6-OH positions to further improve the biological activities of such derivatives.

With the development of the environmentally friendly society, the problems caused by chemical products have attracted people’s attention. Especially, the use of chemical fungicides often causes serious environmental pollution [20,21,22]. Moreover, their toxicities and residues give rise to serious problems for human health [23]. More and more effective fungicides have been prohibited in recent years and it is imperative to explore new chemical bacteriostat, which not only can restrain the growth of the microorganism effectively, but also be biodegradable and environmentally friendly [24]. Thus, the use of novel polysaccharide derivatives could be a promising handling as antibacterial agents to partially substitute for the harmful synthetic bactericides and fungicides.

In the current study, we tried to modify 3-OH and 6-OH of chitin and then 6-amino-chitin (NCT) and 3,6-diamino-chitin (DNCT) were successfully synthesized. The structures of the chitin derivatives were assessed by elemental analyses, FT-IR and ^13^C NMR. In additional, six plant-threatening fungi, including *Fusarium oxysporum* f. sp. *cucumerium* (*F. oxysporum* f. sp. *cucumerium*), *Botrytis cinerea* (*B. cinerea*), *Colletotrichum lagenarium* sp. *cucumerium* (*C. lagenarium*), *Phomopsis asparagi* (*P. asparagi*), *Fusarium oxyspirum* f. *niveum* (*F. oxyspirum* f. *niveum*), and *Gibberella zeae* (*G. zeae*), were selected to evaluate the antifungal property of NCT and DNCT.

## 2. Results and Discussion

### 2.1. Chitin Derivatives Preparation and Characterization

#### 2.1.1. Synthesis of Chitin Derivatives and Elemental Analysis

**Scheme 1:**
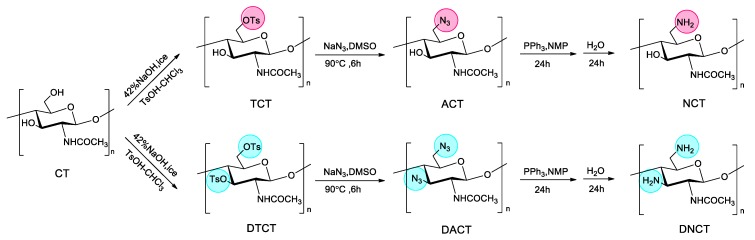
Synthetic route for the preparation of 6-amino-chitin (NCT) and 3,6-diamino-chitin (DNCT).

6-amino-chitin (NCT) and 3,6-diamino-chitin (DNCT) were synthesized as shown in Scheme 1. There are two hydroxyl groups in glucopyranose unit of chitin, a primary hydroxyl at C-6 and a secondary hydroxyl at C-3. Generally, the substitution reaction takes place mostly at the C6-OH rather than C3-OH. The primary hydroxyl at C-6 is the higher chemical reactive due to the lack of intramolecular hydrogen bonds with neighboring repeating units and the lack of steric hindrance against neighboring acetamide groups. In this study, C6-amino-chitin and C3,6-diamino-chitin were obtained by controlling the reaction time and equivalents of reagents. The reaction process is as follows: firstly, 6-tosyl-chitin (TCT) intermediate was prepared through the tosylation of C6-OH group since the tosyl group is a good electrophile and leaving group. When the reduced pressure time was extended to 5 h and the amount of tosyl chloride was doubled, 3,6-ditosyl -chitin (DTCT) was also obtained. Then, the resulting TCT and DTCT can serve as a useful intermediate to react with NaN_3_ in DMSO to produce 6-azido-chitin (ACT) and 3,6-diazido-chitin (DACT). Lastly, 6-amino-chitin (NCT) and 3,6-diamino-chitin (DNCT) were synthesized through the reduction reaction of PPh_3_. The final product, NCT and DNCT were insoluble in water, acetic acid, DMSO, DMF, etc. The abbreviations for the derivatives are presented in Table 1.

The elemental analysis and degree of substitution (DS) of chitin derivatives are presented in Table 2. The degree of substitution of TCT is 0.93, which means that there are 93% of hydroxyl groups at C-6 being displaced by tosyl groups. The DS of DTCT is 1.2 based on elemental analysis, i.e., the C3-OH moiety was partially substituted, which was consistent with the literature [25]. Accurately, in DTCT, 3,6-ditosyl-chitin accounts for 20% and 6-tosyl-chitin for the remaining 80% by calculation. Since the tosyl groups have many carbon and hydrogen atoms as well as the molecular structures of TCT and DTCT are different, it could also happen that the proportion of nitrogen in DTCT is lower than that in TCT. Because the first step could affect the latter reaction, it makes sense that the proportion of nitrogen in DACT is 13.3% while that of ACT is 15%. However, the DS of DACT is >1, which confirmed the substitution of NH_2_ in position of C3 and C6.

#### 2.1.2. FT-IR Spectra

**Figure 1:**
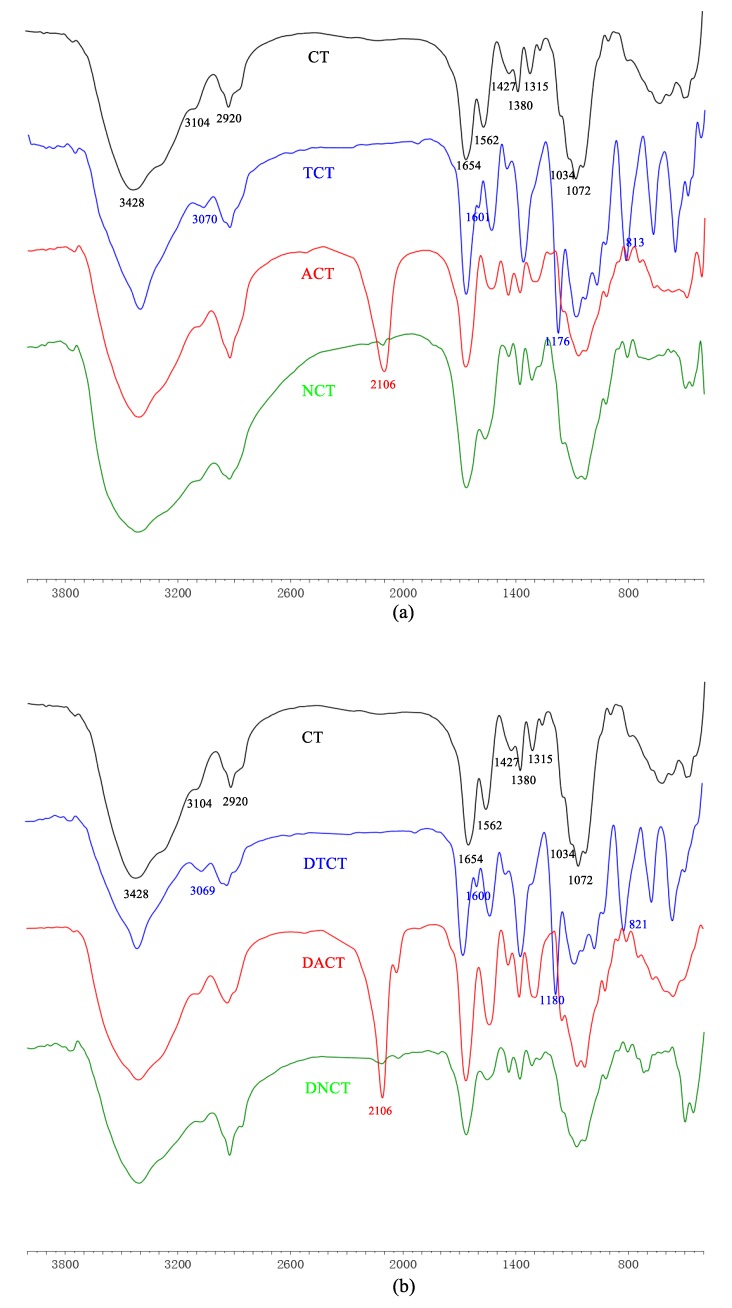
FT-IR spectra of chitin, its monosubstituted (**a**) and disubstituted (**b**) derivatives.

The FT-IR spectroscopy has been demonstrated to be a powerful tool for studying the physicochemical properties of polysaccharide. The FT-IR spectra of chitin and its derivatives are shown in Figure 1. The spectra of the monosubstituted chitin derivatives are similar to those of the disubstituted chitin derivatives.

For chitin, peaks of saccharide were observed at 3428 cm^−1^, 3104 cm^−1^, 2920 cm^−1^, 1654 cm^−1^, 1562 cm^−1^, 1315 cm^−1^, 1427cm^−1^, 1380 cm^−1^, 1072 cm^−1^, and 1034 cm^−1^. The peaks at approximately 3428 cm^−1^ and 3104 cm^−1^ indicated the presence of -OH and -NH of chitin. The band at 2920 cm^−1^ was assigned to the -CH stretching vibration. The peaks at 1654 cm^−1^, 1562 cm^−1^, and 1315 cm^−1^ could be attributed to deformation of amide I, amide II, and amide III, respectively [4]. The other peak at 1427cm^−1^ was corresponded to the absorption of pyranose ring [26]. Moreover, characteristic peak of the acetamide groups appeared at 1380 cm^−1^. The strong absorptions at around 1072 cm^−1^ and 1034 cm^−1^ were related to vibrations of C–O of secondary hydroxyl group and primary hydroxyl group, respectively.

For TCT and DTCT, new strong peaks appeared at 3070 cm^−1^, 1176 cm^−1^, 1601 cm^−1^, and 813 cm^−1^, which were assigned to the stretching vibration of tosyl CH, SO_2_, aromatic C=C, and phenylene, respectively, on tosyl groups, indicating the success of tosyl substitution [25].

After azidation of the chitin derivatives, the peaks at 1176 cm^−1^, 813 cm^−1^, and 1601 cm^−1^ disappeared due to the leaving of tosyl groups. Meanwhile, there was a new strong peak at 2106 cm^−1^, which could be attributed to the characteristic absorption of CN_3_ in the molecule of ACT or DACT.

For NCT and DNCT, the strong peak at 2106 cm^−1^ disappeared when C-N_3_ was reduced to C-NH_2_. In addition, the weakening of peaks at 1032 cm^-1^ and 1068 cm^−1^ proved that the reactions occurred at primary and secondary hydroxyl groups of chitin as well as 6-amino-chitin and 3,6-diamino-chitin were obtained successfully.

#### 2.1.3. Solid-State ^13^C NMR Analysis

**Figure 2:**
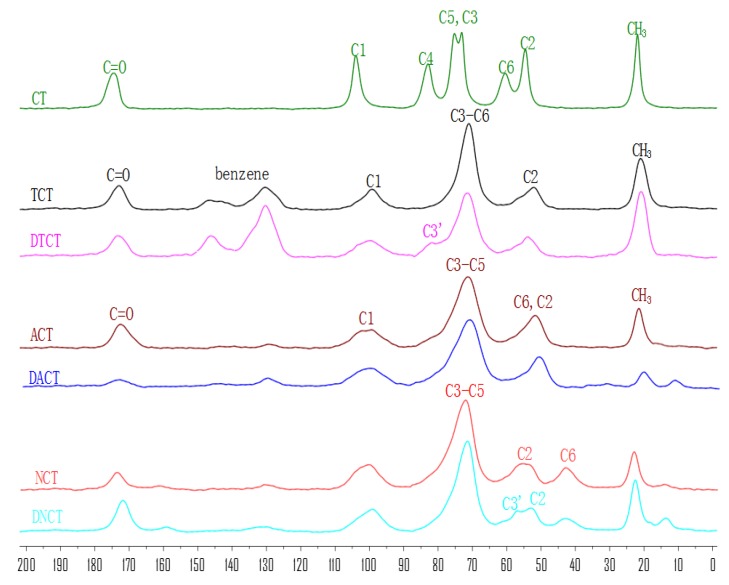
The solid-state ^13^C NMR spectra of chitin, its monosubstituted derivatives and its disubstituted derivatives.

Solid-state ^13^C NMR spectroscopy has been proven to be the most useful method in analyzing the structure of insoluble biopolymers without destroying their conformation [27]. This technique was used to obtain the structural information of chitin and its derivatives (Figure 2).

In the ^13^C NMR spectrum of CT, the signals at 104 ppm, 83 ppm, 76 ppm, 74 ppm, 61 ppm, and 55 ppm were assigned to C1, C4, C5, C3, C6, and C2, respectively [28,29]. In addition, the CH_3_ group of the acetyl moiety gave a signal at 23 ppm, while its carbonyl group produced a peak at 173 ppm.

As to the spectra of TCT, there were new signals at 145–130 ppm, which were related to chemical shift of benzene ring. Meanwhile, the peak of C6 at 61 ppm shifted to lower field (71 ppm) after tosyl substitution, which suggested that hydroxyl groups on C6 had been substituted with tosyl groups. In the spectra of DTCT, new signal appeared at 82 ppm since the C3-OH moiety was partially substituted with tosyl groups and it indicated the successful synthesis of 3,6-ditosyl-chitin.

In the spectra of ACT and DACT, the peaks of the tosyl groups disappeared and the signals of C3 and C6 shifted to 72 ppm and 52 ppm, which led to the conclusion that the conversion from OTs to N_3_ took place.

In the spectrum of NCT, there was a broad peak at 45 ppm, which was assigned to the absorption of C6-NH_2_, suggesting that the amino groups were successfully incorporated into the C6 position of chitin. However, for DNCT, new peaks at 57 ppm and 43 ppm appeared and they could be attributed to signals of C3NH_2_ and C6-NH_2_, respectively. These indicated that the amino groups were successfully incorporated at the C3 and C6 position of CT.

#### 2.1.4. X-ray Diffraction (XRD) Analysis

**Figure 3:**
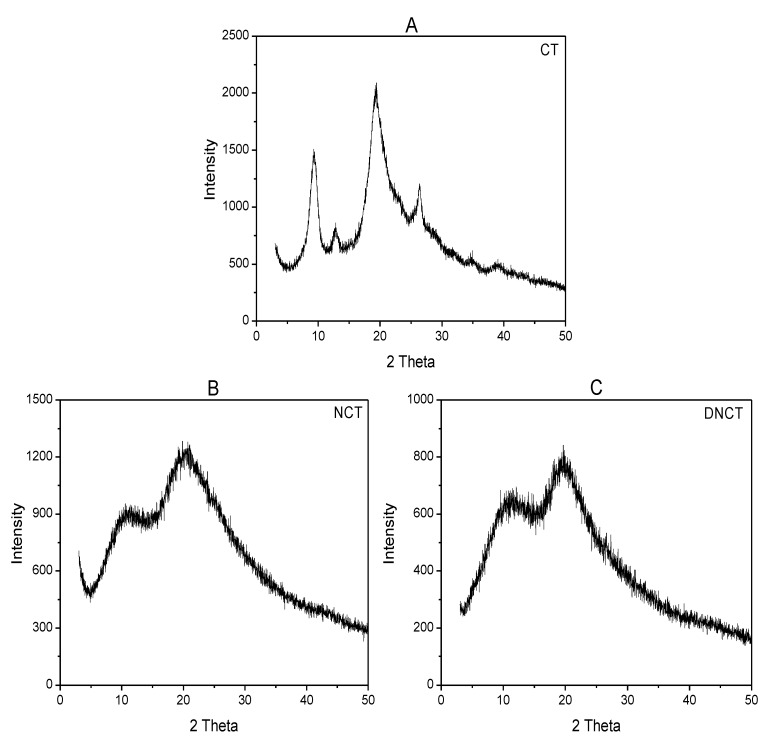
XRD spectra of chitin (**A**), 6-amino-chitin (**B**), and 3,6-diamino-dideoxychitin (**C**).

The X-ray diffraction pattern for CT, NCT, and DNCT were given between 5° and 60° of 2θ in Figure 3. CT peaks were observed at 9.3°, 12.8°, 19.4°, and 25.3°. Two of them are sharp (9.3° and 19.4°) and the other two are faint [6,30]. Compared with CT, the XRD patterns of NCT and DNCT exhibited some changes in their angles, peak intensity, and peak width. The sharp peak of CT at 9.3° shifted to 11.0° and 11.2° in the patterns of NCT and DNCT. Similarly, the sharp peak at 19.4° shifted to 19.6° and 19.8° in the patterns of NCT and DNCT. Both sharp peaks of NCT and DNCT were apparently broader than those of CT. Meanwhile, intensity of both peaks decreased along with the disappearance of both peaks (12.8° and 25.3°). The disappearance peaks of both may be due to the destruction of hydrogen bonding [31].

It is well-known that the width of X-ray diffraction peak is related to the size of crystallite and the broadened peak usually results from small crystallite [32]. In addition, weaker peak implies that there is more amorphous phase in the matrix. From the spectra, we concluded that the substitution might have led to the decrease of crystallinity which could be seen in the form of relatively weaker reflection and broader width in the spectra of NCT and DNCT. The higher substitution, the lower crystallinity. Moreover, substitution also resulted in the changes of symmetry and step regularity.

#### 2.1.5. Scanning Electron Microscopy (SEM) Analysis

**Figure 4:**
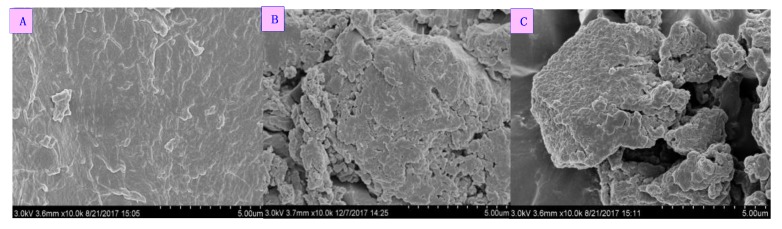
Surface morphology of chitin (**A**), 6-amino-chitin (**B**), and 3,6-diamino-chitin (**C**).

The morphology of CT, NCT, and DNCT were studied by scanning electron microscopy (SEM) (Figure 4). CT exhibited compact and flat morphology, while NCT and DNCT showed loose and porous structures. According to the results, we speculated that the morphology structure of CT changed as a consequence of the formation of a chitin derivative. It was concluded that loose and porous structure result in weaker thermal stability of NCT and DNCT.

### 2.2. Antifungal Activity

Some data on the average inhibition diameters of CT, NCT, and DNCT are given in Table 3.

According to the results, the negative control treatments (deionized water) had no inhibitory effect on any of the test fungi. Similarly, CT had little antfungal activity. On the contrary, carbendazim showed good inhibition on all of the tested fungi and inhibition zone diameters were more than 16.3 mm. Meanwhile, another positive control, amphotericin B, showed weaker antifungal activity than that of carbendazim. Surprisingly, NCT and DNCT possessed excellent fungal activity. The inhibition zone diameters of NCT varied between 11.2 and 16.3 mm and the inhibition zone diameters of DNCT varied between 11.4 and 20.4 mm. NCT and DNCT had the highest inhibition zone values against *F. oxysporum* f. sp. *cucumerium* (16.3 mm and 21.4 mm). In addition, compared to amphotericin B, NCT showed better inhibitory activity against two fungi, and DNCT showed better inhibitory activity than amphotericin B against three fungi.

There was a relationship between the antibacterial or antifungal activity of polysaccharide and the free amino groups. The greater the number of amino groups in the polysaccharide derivatives, the better their antifungal activity was [19,33], which confirmed the crucial role of amino groups in enhancing the antifungal activity of chitin. For this reason, NCT and DNCT have more amino compared to CT, which could be expected to have a more potent antifungal activity. The observation is also in agreement with the results obtained by Ren, who found that carbohydrate derivatives with greater amino groups showed better antifungal activity against the plant pathogenic fungi.

## 3. Materials and Methods

### 3.1. Materials

CT was supplied by Sinopharm Chemical Reagent Co., and deacetylation degree (DD) of 10%. The DD was determined by elemental analysis. The other reagents such as dimethyl sulfoxide (DMSO), sodium azide, triphenylphosphine (Ph_3_P), acetic anhydride, trichloromethane, 4-toluene sulfonyl chloride, etc., were supplied by Sinopharm Chemical Reagent Co., Ltd. (Shanghai, China).

### 3.2. Analytical Methods

FT-IR spectra were measured on a Jasco-4100 Fourier Transform Infrared Spectrometer (JASCO Co., Ltd., Shanghai, China) with KBr disks. The elemental analyses (C, H, S, and N) were performed on a Vario Micro Elemental Analyzer (Elementar, Berlin, Germany). The degrees of substitution (DS) were calculated on the basis of the percentages of carbon and nitrogen. The UV-Vis absorbance was measured with a T6 New Century UV spectrometer (P General Co., Ltd., Beijing, China). ^13^C CP/MAS NMR experiments were performed on Bruker AVANCE III 600 spectrometer (Bruker Tech. and Serv. Co., Ltd., Beijing, China) at a resonance frequency of 150.9 MHz. ^13^C CP/MAS NMR spectra were recorded using a 4 mm MAS probe and a spinning rate of 12 kHz. The X-ray diffraction patterns of samples were recorded at room temperature on an X-ray diffractometer (D8 advance, Bruker, Berlin, Germany). The morphology of the samples was examined through a Scanning electron microscope (SEM) (S-4800, Hitachi, Tokyo, Japan). Each sample was coated with gold in an ion sputter (E-1045, Hitachi, Tokyo, Japan) before being scanned and photographed at the magnifications (1000×). An accelerating potential of 3 kV was used during image acquisition.

### 3.3. Synthesis of Chitin Derivatives

#### 3.3.1. Tosylation of Chitin [25]

Preparation of 6-tosyl-chitin (TCT)

A mixture of 1.0 g of chitin and 20 mL of 42 % aqueous sodium hydroxide was left standing at a reduced pressure for 2 h. To this was added 50 g of crushed ice, and the mixture was stirred to give a clear solution. It was then cooled in an ice bath, and 7.5 g (7.5 mol equivalent to pyranose rings) of tosyl chloride dissolved in 20 mL of chloroform was added with stirring. After 2 h of reaction, the ice bath was replaced, and the mixture was stirred at room temperature for another 2 h. The mixture was gradually poured into water, and the precipitate was washed with water until neutral. Then the precipitate was washed with ethanol and dried at 60 °C. The product was obtained as a white solid.

Preparation of 3,6-ditosyl-chitin (DTCT)

The synthesis method of DTCT is similar to that of TCT, except that the reduced pressure time was extended to 5 h and the amount of tosyl chloride was doubled.

#### 3.3.2. Azidation of Tosyl Chitin

Preparation of 6-azido-chitin (ACT) and 3,6-diazido-chitin(DACT)

A mixture of 2.5 g tosylation of chitin and 80 mL DMSO was treated with sodium azide (3.6 g) at 90 °C for 6 h. The resulting mixture was cooled to room temperature and then poured into 400 mL ethyl acetate. The precipitate was collected, washed with ethanol, and then dried at 60 °C to give the product.

#### 3.3.3. Reduction of Azide Group

Preparation of 6-amino-chitin (NCT) and 3,6-diamino-chitin (DNCT)

Azidation of chitin (0.63 g) was suspended in 65 mL of DMF and 2.83 g of triphenylphosphine was added. After 24 h of reaction at room temperature, 1.3 mL water was then added to the mixture. After another 24 h of reaction, the resulting solid was washed with 200 mL of ethanol, and dried to give the product.

### 3.4. Evaluation of Antifungal Activity in Vitro

Antifungal activities of CT, NCT, and DNCT against plant pathogens were investigated by disk diffusion method under in vitro condition [34]. Carbendazim and amphotericin B were used as positive controls. Deionized water was used as a negative control. Firstly, autoclaved (sterile) fungi medium was prepared. Then, it was poured into sterile plates and cultured media were allowed to solidify. 0.1 mL *F. oxysporum* f. sp. *cucumerium*, *B. cinerea*, *C. lagenarium*, *P. asparagi*, *F. oxyspirum* f. *niveum*, and *G. zeae* were inoculated on the fungi medium respectively, and then spread on the entire surface of the medium by sterile spatula. Next, the well of 5 mm diameter was bored using a sterile borer in each plate. In each well, 5 mg of samples were loaded. The fungal plates were incubated at 27 °C and zones of inhibition was measured after 3 days. Greater inhibition zone indicated higher antifungal activity.

### 3.5. Statistical Analysis

Data were analyzed by the analysis of variance to guarantee statistical significance. All data were expressed as means ± standard deviation. The Scheffe method was used to evaluate the differences in antifungal index in the antifungal assays. The *p*-values of less than 0.05 were considered to be statistically significant and results were analyzed by Excel.

## 4. Conclusions

In summary, 6-amino-chitin and 3,6-diamino-chitin were synthesized successfully via tosyl-chitin. The antifungal activity of NCT and DNCT against six plant fungi were tested, and satisfactory results were obtained. Firstly, NCT and DNCT showed stronger antifungal activity than CT in all tests. Secondly, compared to amphotericin B, NCT showed better inhibitory against three fungi and DNCT showed better inhibitory against four fungi. Finally, the order of the inhibitory activity, which is DNCT > NCT > CT, suggested that amino group was the major factor in antifungal activity and NCT and DNCT might be a potential effective antifungal agent. The development of fungicides has attracted people’s attention along with the food safety. New environment-friendly effective substitutes of chemical fungicides need to be explored. These findings mentioned above bring further evidence that chitin derivatives are active and have the potential of becoming alternatives of some harmful pesticides for disease control.

## Figures and Tables

**Table 1:** The abbreviations for the derivatives.

Monosubstituted Chitin Derivatives		Disubstituted Chitin Derivatives	
Full Name	Abbreviations	Full Name	Abbreviations
6-tosyl-chitin	TCT	3,6-ditosyl-chitin	DTCT
6-azido-chitin	ACT	3,6-diazido-chitin	DACT
6-amino-chitin	NCT	3,6-diamino-chitin	DNCT

**Table 2:** The elemental analyses of chitin (CT), its monosubstituted derivatives, and its disubstituted derivatives.

Sample	Found (%)				DS	Formula
	C	H	N	S		
CT	45.2	7.2	6.6	-	-	C_8_H_13_NO_5_
TCT	50.2	6.6	4.0	8.3	0.93	(C_15_H_19_NO_7_S)_0.93_(CT)_0.07_
DTCT	50.3	13.9	3.6	10.0	1.2	(C_15_H_19_NO_7_S)_0.8_(C_22_H_25_NO_9_S_2_)_0.2_
ACT	43.3	5.3	21.9	-	0.97	(TCT)_0.1_ (C_8_H_12_N_4_O_4_)_0.9_
DACT	40.4	4.7	21.3	-	1.14	(DTCT)_0.03_(C_8_H_12_N_4_O_4_)_0.78_ (C_8_H_11_N_7_O_3_)_0.18_
NCT	47.0	6.8	15.0	-	0.96	(ACT)_0.04_ (C_8_H_14_N_2_O_4_)_0.87_
DNCT	45.5	6.4	13.3	-	1.11	(DACT)_0.03_ (C_8_H_14_N_2_O_4_)_0.76_(C_8_H_15_N_3_O_3_)_0.17_

**Table 3:** Antifungal activity against various fungi (from A to F were represented *F. oxysporum* f. sp. *cucumerium*, *B. cinerea*, *C. lagenarium*, *P. asparagi*, *F. oxyspirum* f. *niveum*, and *G. zeae*, respectively).

Sample	Inhibition Zone Diameter Against Various Fungi (mm) ^a^					
	A	B	C	D	E	F
Carbendazim	24.3 ± 1.3	19.9 ± 0.9	16.3 ± 0.5	27.8 ± 1.1	18.6 ± 0.4	21.8 ± 1.0
Amphotericin B	16.1 ± 1.0	14.2 ± 0.4	8.0 ± 0.8	18.9 ± 1.8	9.6 ± 0.5	15.7 ± 0.7
CT	5.3 ± 0.1	5.5 ± 0.2	5.3 ± 0.1	5.8 ± 0.2	5.8 ± 0.2	6.1 ± 0.2
NCT	16.3 ± 1.3 *	11.2 ± 0.5 **	13.1 ± 0.9 *	14.2 ± 1.0 *	12.4 ± 0.2 **	14.9 ± 0.6 **
DNCT	20.4 ± 1.7 **	11.4 ± 0.2 **	14.0 ± 0.5 **	15.6 ± 1.5 *	14.2 ± 0.5 **	16.3 ± 1.3 **

^a^ Zone diameter of the inhibition including disc. Values are presented as means ± SD of three separate experiments (*: 0.01 < P < 0.05; **: 0.001 < P < 0.01).
